# Supplementary material for: Semiarid climate and hyposaline lake on early Mars inferred from reconstructed water chemistry at Gale
Source: Nat Commun. 2019 Oct 25;10:4896. doi: 10.1038/s41467-019-12871-6 (PMC6814795; doi:10.1038/s41467-019-12871-6)
Supplement: Supplementary file 1 — Supplementary Information [file 41467_2019_12871_MOESM1_ESM.docx]

**Supplementary Information for**

**Semiarid climate and hyposaline lake on early Mars inferred from reconstructed water chemistry at Gale**

by Fukushi et al.

Supplementary Figures

**Supplementary Fig. 1 Schematic diagram showing the cation exchange reaction of smectite.** The rate of the exchange reaction is sub-second order with low activation energy. In the presence of liquid water, the cation exchange reactions between the solution and the interlayer of smectite occur instantaneously even in the low temperature conditions. The interlayer cation composition in smectite after the disappearance of the surrounding liquid water records the cationic composition of liquid water just before the disappearance.

**Supplementary Fig. 2 Results of dissolved components in the pore water of Yellowknife Bay sediments for the full-interaction and no-interaction scenarios.**

**Supplementary Fig. 3 Pictures of hyposaline lakes in semiarid regions on Earth.** a,b) Shores of the Lonar crater lake, India (salinity of 0.1–0.2 mol/kg), c,d) Shores of the Böön Tsagaan lake (salinity of 0.05–0.1 mol/kg), Mongolia, and e) Shore of the Orog lake (salinity of 0.05–0.1 mol/kg), Mongolia. Desiccation cracks can be seen in the panels a) and b), but they are not pervasive and small (width 0.3–1 m) surrounding the lake. The panels f) and g) show the locations of the Lonar Crater, and the Böön Tsagaan lake and Orog lake, respectively. The maps were created by tracing the satellite images (Google Earth). The points a)–e) in the satellite images in the panels f) and g) represent the locations where the above images were taken.

**Supplementary Fig. 4 Decomvolution of the (001) reflection of smectite from XRD pattenrs from Cumberland site and John Klein site.** The atomic structure factors of each cationic component in the interlayer can be assumed to beare unity. The assumption wais verified from the computer simulation of the intensities of 001 reflections of each cationic component with and without the hydration effects by using NEWMOD program^1^. The LP factor of the XRD patterns of John Klein and Cumberland were provided with courtesy from E.B. Rampe (personal communication). The Lorentz-polarization factors and the backgrounds were corrected from the original XRD patterns.


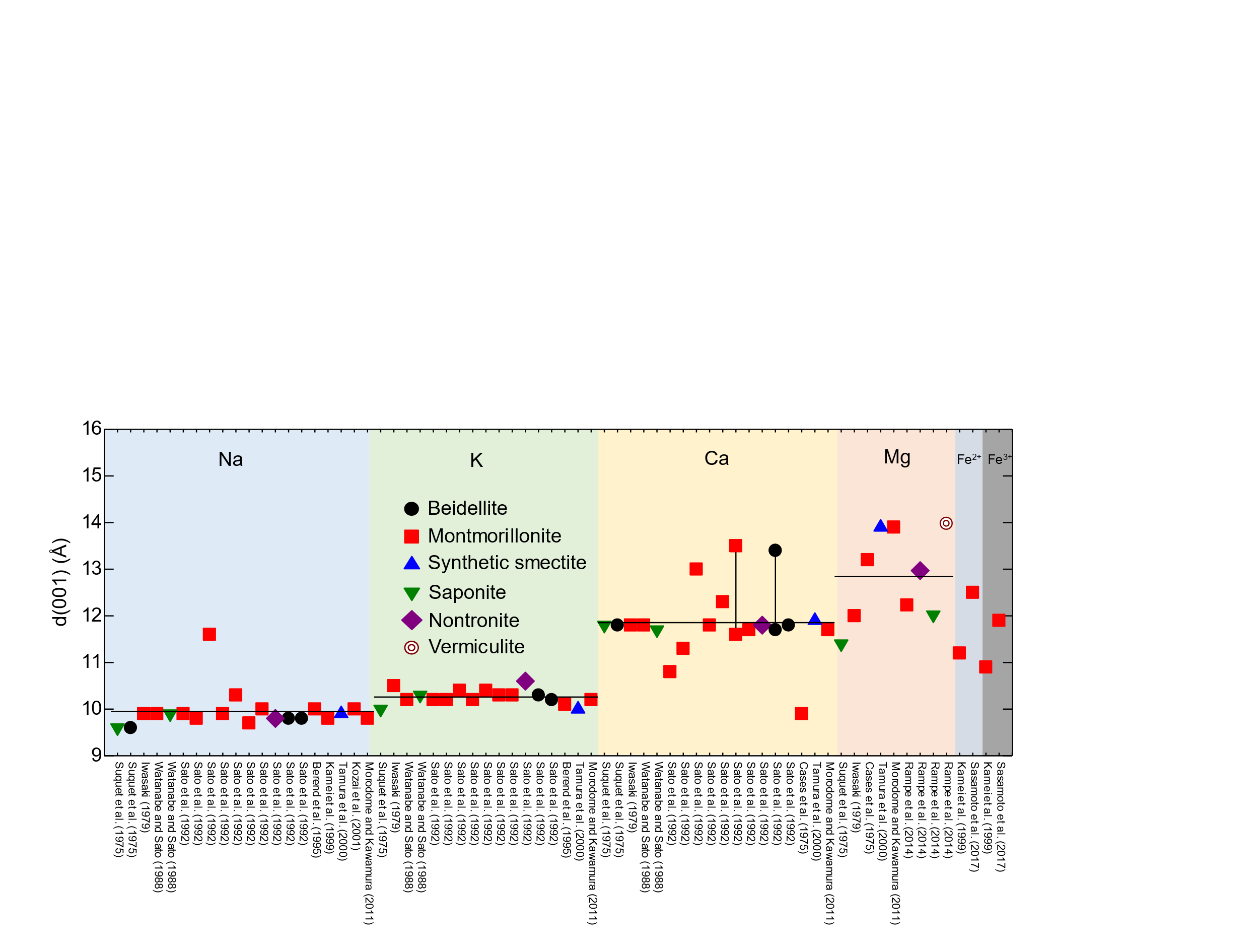


**Supplementary Fig. 5 Summary of examinations of the basal spacings of smectite and vermiculite with different cations (Na, K, Ca, Mg, Fe^2+^ and Fe^3+^) under RH = 0% (Ref^2–13^).** The data were ordered according to the published year for each cation.

**Supplementary Fig. 6 Results of re-analysis of a three-dimensional general circulation model (GCM) for a cold and 1-bar case performed by the previous study**^14^**.** a.) Global distribution of annual mean surface wind speed. The unit for the color bar is m/sec. The red point shows the location of Gale Crater b.) Time series of wind surface wind speed at the location of Gale Crater (3.75° S and 135 E. in the gridpoint in the GCM).

Supplementary Tables

**Supplementary Table 1. Equivalent ratios of the cationic composition from John Klein and Cumberland sites given by ${\boldsymbol{\beta}_{\boldsymbol{j}}}$ in Eq. (3) and the selectivity coefficients ($K_{\text{Na\_}i}$) based on the generic empirical model**^15^

**Supplementary Table 2. Mole ratio of major cations in nature water contacting with saponite in terrestrial condition (Ref.** ^16–22^**).**

Supplementary Notes

**Supplementary Note 1**

**Equilibrium constants for the dissolution reactions of Fe-saponite, akaganeite and ferrihydrite** Mass balance calculations using bulk-rock chemical data from APXS and the chemistry of crystalline components indicate that the mole ratios of Mg to Fe of the clay minerals and amorphous materials were comparable for both of the John Klein and Cumberland samples^23^. Since the interlayer cation compositions of the smectite are dominated by Na^+^ or Mg^2+^ (see main text), the chemical composition of Fe^2+^-saponite was assumed to be Na_0.175_Mg_0.0875_Fe_1.5_Mg_1.5_Al_0.35_Si_3.65_O_10_OH_2_. The solubility of the Fe^2+^-saponite comes from the estimation by Wilson et al.^24^.

Biedermann and Chow^25^ examined the hydrolysis of Fe(III) species in 0.5 M NaCl solutions at 25 ºC. They found that the precipitates formed during the hydrolysis experiments were akaganeite from the X-ray diffraction analysis. They observed the following linear relationship between the H^+^ and Fe^3+^ concentrations under several different initial Fe(III) concentrations:

2.70 log [H^+^] – log [Fe^3+^] = –3.04 ± 0.05 (19)

where [*i*] denote the molar concentration of *i*th species. According to the coefficient of log [H^+^], the chemical formula of akaganeite is considered to be FeO(OH)_0.7_Cl_0.3_. The dissolution reaction and the mass action expression of the akaganeite can be written:

FeO(OH)_0.7_Cl_0.3_ + 2.7H^+^ = Fe^3+^ + 0.3Cl^-^ +1.7H_2_O (20)

$K_{akaganeite}=\frac{a_{\text{F}e^{3+}}a_{\text{Cl}^{\text{-}}}^{0.3}a_{\text{H}_{2}\text{O}}^{1.7}}{a_{\text{H}^{+}}^{2.7}}=\frac{m_{\text{F}e^{3+}}}{m_{\text{H}^{+}}^{2.7}}\frac{\gamma_{\text{F}e^{3+}}}{\gamma_{\text{H}^{+}}^{2.7}}a_{\text{Cl}^{\text{-}}}^{0.3}a_{\text{H}_{2}\text{O}}^{1.7}$ (21)

where $a_{i}$,$m_{i}$and$\gamma_{i}$represent activity, molal concentration and activity coefficient of *i*th species. Assuming [H^+^] and [Fe^3+^] correspond to$m_{\text{H}^{\text{+}}}$and$m_{\text{Fe}^{\text{3+}}}$because of the low concentrations (mostly <0.01M) of the species in the solution, Eq. (21) can be written:

$K_{akaganeite}=\frac{a_{\text{F}e^{3+}}a_{\text{Cl}^{\text{-}}}^{0.3}a_{\text{H}_{2}\text{O}}^{1.7}}{a_{\text{H}^{+}}^{2.7}}=10^{3.04}\frac{\gamma_{\text{F}e^{3+}}}{\gamma_{\text{H}^{+}}^{2.7}}a_{\text{Cl}^{\text{-}}}^{0.3}a_{\text{H}_{2}\text{O}}^{1.7}$ (22)

Other variables in Eq. (22) was calculated with the REACT program with the same activity model under 0.1 mol/L NaCl solution. The equilibrium constant for the akaganeite dissolution was estimated to be $K_{akaganeite}=$1.61± 0.05.

Mazeina et al.^26^ obtained the standard enthalpy of formation of akaganeite as -554.7 ± 1.9 kJ/mol. The equilibrium constant of akaganeite at 0 ºC was estimated from van’t Hoff equation:

$\ln\frac{K_{akaganeite,0}}{K_{akaganeite,25}}=\frac{-\Delta H_{R}^{0}}{R}(\frac{1}{273+0}-\frac{1}{273+25})$ (23)

The $\Delta H_{R}^{0}$ based on the reaction in Eq. (20) was estimated from the standard enthalpy of formation of akaganeite and the standard enthalpy of formation of the relevant species from the “thermo.dat” dataset in the Geochemist’s Workbench^27^. The equilibrium constant for the akaganeite dissolution at 0 ºC was estimated to be $K_{akaganeite,0}=$2.11± 0.08.

Hiemstra^28^ examined the solubility and the standard enthalpy of formation of ferrihydrite from the theoretical consideration and the comprehensive review of the previous studies. He showed that the solubility products of the 2-line ferrihydrite is log K_SO_ = -38.5± 0.1 assuming following dissolution reaction:

FeO_3/2_ + 3/2 H_2_O = Fe^3+^ + 3OH^-^ (21)

The standard enthalpy of formation of ferrihydrite with typical range of the specific surface area (400 – 800 m^2^/g) is 400 ± 2 kJ/mol (FeO_3/2_). Present study assumes the following dissolution reaction of ferrihydrite during the calculation in REACT:

Fe(OH)_3_ + 3H^+^ = Fe^3+^ + 3H_2_O (22)

Based on the solubility product and the standard enthalpy of formation estimated by Hiemstra^28^, the equilibrium constants at 0 ºC was estimated to be log$K_{ferrihydrite,0}=$4.8± 0.1.

**Supplementary Note 2**

**Duration estimate for Lonar lake** The present study evaluates our methodology to estimate the duration of a closed-basin lake from its salinity. An ideal terrestrial analog that allows us to test our methodology is Lonar lake, which is within an impact structure formed upon Deccan basaltic rocks, India (lake diameter = 1.2 km, mean lake depth = 5 m)^29–31^. Given seasonal variations in lake levels (< 2 m)^32^ and the annual precipitation of ~700 mm/year in semiarid climates^33^, the annual evaporation rate would be a few meters/year in lake level, which corresponds to ~2 × 10 kg/sec of *F*_ground_. Spring waters at Lonar contain low Na of ~10^-3^ mol/kg (*C*_ground_)^30^. Although Lonar lake is hyposaline at present (Na^+^ ~0.2 mol/kg), Na concentrations of the lake in 1910 were ~2 mol/kg (ref. ^31^). The decline in salinity over time is, at least partly, because of the influence of humans near the lake^31^. Thus, we use ~2 mol/kg for the original Na concentration of the lake (*C*_lake_). Introducing these values into Eq. (18), *t* becomes (1–2) × 10^4^ years. The duration may be shorter than the proposed crater age, (1–5) × 10^4^ years^30,34,35^. This could be because the occurrence of Na^+^ precipitation as gaylussite in the sediments during arid periods (4,600–3,900 and 2,000–600 cal a BP)^32^, and because climate changes during the Holocene. Despite these factors, the estimated duration is consistent with the crater age within a factor of 5. This suggests our methodology to estimate lake duration within an order of magnitude is valid, even with the occurrence of repeated progressive evaporations and climate change.

**Supplementary References**

1. Walker, J. R. An Introduction to Computer Modeling of X-Ray Powder Diffraction Patterns of Clay Minerals: A Guided Tour of NEWMOD©. *Computer Applications to X-Ray Powder Diffraction Analysis of Clay Minerals* **5**, 1–17 (1993).

2. Morodome, S. & Kawamura, K. In situ X-ray diffraction study of the swelling of montmorillonite as affected by exchangeable cations and temperature. *Clays Clay Miner.* **59**, 165–175 (2011).

3. Sato, T., Watanabe, T. & Otsuka, R. Effects of layer charge, charge location, and energy change on expansion properties of dioctahedral smectites. *Clays Clay Miner.* **40**, 103–113 (1992).

4. Rampe, E. B. *et al.* Characterizing the phyllosilicate component of the Sheepbed mudstone in Gale Crater, Mars using laboratory XRD and EGA. *45th Lunar Planet. Sci. Conf.* **2**, 2–3 (2014).

5. Sasamoto, H., Isogai, T., Kikuchi, H., Satoh, H. & Svensson, D. Mineralogical , physical and chemical investigation of compacted Kunigel V1 bentonite in contact with a steel heater in the ABM test package 1 experiment , Äspö laboratory , Sweden. *Clay Miner.* **52**, 127–141 (2017).

6. Suquet, H., de la Calle, C. & Pezerat, H. Swelling and Structural Organization. *Clays Clay Miner.* **23**, 1–9 (1975).

7. Iwasaki, T. Relationship between X-ray basal reflections and interlayer cations of montmorillonite: on the distribution of Ca and Na ions. *J. Mineral. Soc. Japan* **14**, 78–89 (1979).

8. Watanabe, T. & Sato, T. Expansion characteristics of montmorillonite and saponite under various relative humidity conditions. *Clay Sci.* **138**, 129–138 (1988).

9. Bérend, I. *et al.* Mechanism of Adsorption and Desorption of Water Vapor by Homoionic Montmorillonites: 2. The Li^+^, Na^+^, K^+^, Rb^+^, and Cs^+^-exchanged Forms. *Clays Clay Miner.* **43**, 324–336 (1995).

10. Kamei, G., Oda, C., Mitsui, S., Shibata, M. & Shinozaki, T. Fe(II)-Na ion exchange at interlayers of smectite: Adsorption-desorption experiments and a natural analogue. *Eng. Geol.* **54**, 15–20 (1999).

11. Tamura, K., Yamada, H. & Nakazawa, H. Stepwise hydration of high-quality synthetic smectite with various cations. *Clays Clay Miner.* **48**, 400–404 (2000).

12. Kozai, N. *et al.* Characterization of fe-montmorillonite: A simulant of buffer materials accommodating overpack corrosion product. *J. Nucl. Sci. Technol.* **38**, 1141–1143 (2001).

13. Cases, J. M. *et al.* Mechanism of adsorption and desorption of water vapor by homoionic montmorillonite. 3. The Mg^2+^,Ca^2+^,Sr^2+^ and Ba^2+^ exchanged forms. *Clays Clay Miner.* **45**, 8–22. (1997).

14. Wordsworth, R. D., Kerber, L., Pierrehumbert, R. T., Forget, F. & Head, J. W. Comparison of “warm and wet” and “cold and icy” scenarios for early Mars in a 3‐D climate model. *J. Geophys. Res. Planets* **120**, 1201–1219 (2015).

15. Tournassat, C. *et al.* Cation Exchange Selectivity Coefficient Values on Smectite and Mixed-Layer Illite/Smectite Minerals. *Soil Sci. Soc. Am. J.* **73**, 928–942 (2008).

16. Darragi, F. & Tardy, Y. Authigenic trioctahedral smectites controlling pH , alkalinity, silica and magnesium concentrations in alkaline lakes. *Chem. Geol.* 59–72 (1987).

17. Yuretich, R. F. & Cerling, T. E. Hydrogeochemistry of Lake Turkana , Kenya: Mass baIance and mineral reactions in an alkaline lake. *Geochim. Cosmochim. Acta* **47**, 1099–1109 (1983).

18. Felmy, A. R. & Weare, J. H. The prediction of borate mineral equilibria in natural waters : Application to Searles Lake , California. *Geochim. Cosmochim. Acta* **50**, 2771–2783 (1986).

19. Hover, V. C., Walter, L. M., Peacor, D. R. & Martini, A. M. Mg-smectite authigenesis in a marine evaporative environment, Salina Ometepec , Baja California. *Clays Clay Miner.* **47**, 252–268 (1999).

20. Inoue, A. Formation and transformation of clay minerals under saline, alkaline conditions- a review in special reference to reactions of bentonite and cement pore fluids in high-level radioactive waste repository. *J. Clay Sci. Soc. Japan* **43**, 145–161 (2004).

21. Drever, J. I. *The Geochemistry of Natural Waters: Surface and Groundwater Environments*. (Prentice Hall, 1997).

22. Gérard, E. *et al.* Key Role of Alphaproteobacteria and Cyanobacteria in the Formation of Stromatolites of Lake Dziani Dzaha ( Mayotte, Western Indian Ocean ). *Front. Microbiol.* **9**, 796 (2018).

23. Morrison, S. M. *et al.* Crystal chemistry of martian minerals from Bradbury Landing. *Am. Mineral.* **103**, 857–871 (2018).

24. Wilson, J., Savage, D., Cuadros, J., Shibata, M. & Ragnarsdottir, K. V. The effect of iron on montmorillonite stability. (I) Background and thermodynamic considerations. *Geochim. Cosmochim. Acta* **70**, 306–322 (2006).

25. Biedermann, G. & Chow, J. T. Studies on the hydrolysis of metal ions. Part 57. The hydrolysis of the iron(III) ion and the solubility product of Fe(OH)_2.70_Cl_0.30_ in 0.5 M (Na^+^)Cl^-^ media. *Acta Chem. Scand.* **20**, 1376–1388 (1966).

26. Mazeina, L., Deore, S. & Navrotsky, A. Energetics of bulk and nano-akaganeite, β-FeOOH: Enthalpy of formation, surface enthalpy, and enthalpy of water adsorption. *Chem. Mater.* **18**, 1830–1838 (2006).

27. Bethke, C. M. *The Geochemist’s Workbench Users Guide*. (University of Illinois, 1998).

28. Hiemstra, T. Formation, stability, and solubility of metal oxide nanoparticles: Surface entropy, enthalpy, and free energy of ferrihydrite. *Geochim. Cosmochim. Acta* **158**, 79–198 (2015).

29. Komatsu, G. *et al.* Drainage systems of Lonar Crater, India: Contributions to Lonar Lake hydrology and crater degradation. *Planet. Space Sci.* **95**, 45–55 (2014).

30. Maloof, A. C. *et al.* Geology of Lonar Crater, India. *Bull. Geol. Soc. Am.* **122**, 109–126 (2010).

31. Tambekar, D. H., Pawar, A. L. & Dudhane, M. N. Lonar Lake Water : Past and Present. *Nat. Envion. Pollut. Tech.* **9**, 217–221 (2010).

32. Anoop, A. *et al.* Palaeoenvironmental implications of evaporative gaylussite crystals from Lonar Lake, central India. *J. Quat. Sci.* **28**, 349–359 (2013).

33. Sengupta, S. & Sarkar, A. Stable isotope evidence of dual ( Arabian Sea and Bay of Bengal ) vapour sources in monsoonal precipitation over north India. *Earth Planet. Sci. Lett.* **250**, 511–521 (2006).

34. Storzer, D. & Koeberl, C. Age of the Lonar impact crater, India: First results from fission track dating. in *35th Proc. Lunar & Planetary Sci. Conf.* #1309 (2004).

35. Nakamura, A. *et al.* Formation and geomorphologic history of the Lonar impact crater deduced from in situ cosmogenic ^10^Be and ^26^Al. *Geochemistry, Geophys. Geosystems* **15**, 3190–3197 (2014).
